# Comparison of Hybrid Strategy With Drug‐Coated Balloons Versus Stent‐Only Strategy for Coronary Bifurcation Lesions: A Systematic Review and Meta‐Analysis

**DOI:** 10.1155/cdr/7598895

**Published:** 2026-09-24

**Authors:** Yuyang Cao, Hao Li, Jiayu Zhang, Queyun Sun, Zhao Ma, Sida Jia, Na Xu, Jinqing Yuan, Ying Song

**Affiliations:** ^1^ National Clinical Research Center for Cardiovascular Diseases, State Key Laboratory of Cardiovascular Disease, Fuwai Hospital, National Center for Cardiovascular Diseases, Chinese Academy of Medical Sciences and Peking Union Medical College, Beijing, China, cacms.ac.cn; ^2^ Bayingolin Mongolian Autonomous Prefecture People′s Hospital, Korla, Xinjiang, China

**Keywords:** coronary bifurcation lesions, drug-coated balloon, drug-eluting stent, hybrid strategy

## Abstract

**Background:**

Coronary bifurcation lesions (CBLs) account for approximately 15%–20% of all percutaneous coronary interventions and remain a therapeutic challenge. A hybrid strategy combining drug‐coated balloons (DCBs) for the side branch with drug‐eluting stents (DESs) for the main vessel has emerged as a novel therapy aimed at mitigating complications associated with permanent implants. This study was aimed at systematically evaluating the efficacy and safety of the hybrid strategy compared with a DES‐only strategy (including single‐ and double‐stent strategies) in CBLs.

**Methods:**

Following the PRISMA 2020 guidelines, we systematically searched major databases for randomized controlled trials (RCTs) and observational studies comparing the DES + DCB hybrid strategy with a DES‐only strategy for CBLs. The primary endpoint was major adverse cardiovascular events (MACE). Secondary endpoints included target lesion revascularization (TLR), target vessel revascularization (TVR), late lumen loss (LLL), myocardial infarction (MI), and restenosis rate. Statistical analyses were performed using RevMan 5.4 and StataMP.

**Results:**

We included 13 studies, comprising five RCTs and eight observational studies, with a total of 2186 patients (1030 in the hybrid strategy group and 1156 in the DES‐only group). Compared with the DES‐only strategy, the hybrid strategy was associated with a lower incidence of MACE (OR 0.75; 95% CI 0.57–0.99; *p* = 0.04) and a lower risk of TLR (OR 0.46; 95% CI 0.26–0.79; *p* = 0.005); however, the MACE benefit was of borderline statistical significance. Regarding angiographic endpoints, the hybrid strategy substantially lowered the risk of side branch restenosis (OR 0.13; 95% CI 0.05–0.34; *p* < 0.0001). Side branch LLL was consistently lower with the hybrid strategy across all contributing studies; however, extreme between‐study heterogeneity (*I* ^2^ = 96*%*) precluded meaningful pooling. Subgroup analysis indicated a more pronounced reduction in MACE risk for left main lesions (OR 0.50; 95% CI 0.31–0.81; *p* = 0.005), with extremely low heterogeneity (*I* ^2^ = 0*%*). No statistically significant differences were observed between the two groups with respect to MI or TVR.

**Conclusions:**

For CBLs, the DES + DCB hybrid strategy appears to offer favorable safety and efficacy compared with a stent‐only strategy. The observed benefits likely reflect both the avoidance of a second permanent stent implant and the localized antiproliferative drug delivery by the DCB, collectively reducing side branch restenosis and TLR. These findings support the hybrid approach as a promising alternative to complex double‐stent techniques, although large‐scale RCTs are warranted to confirm these results.

## 1. Introduction

Coronary bifurcation lesions (CBLs) represent one of the most common and challenging subsets of coronary artery disease. Although they account for only 15%–20% of all percutaneous coronary interventions (PCI) [1], the high rate of adverse events is also a matter of intense research and scientific debate. While drug‐eluting stents (DESs) remain the standard of treatment for CBLs [2], the optimal management strategy for the side branch remains controversial. Although double‐stent techniques are sometimes anatomically unavoidable, complex metallic layering increases the risk of in‐stent restenosis (ISR) and thrombosis, particularly in elderly patients and those at high bleeding risk (HBR). Therefore, there is an urgent clinical need to explore more effective therapeutic regimens to minimize these complications.

Drug‐coated balloons (DCBs) offer a potential alternative. By applying a drug coating to the balloon surface to exert anti‐intimal hyperplasia effects [3], they provide antiproliferative drug delivery without leaving a permanent implant. This approach can reduce the risk of late complications while preserving native vessel geometry and physiological function. Thus, DCBs are considered a promising option for treating CBLs, aligning with the evolving “leave nothing behind” concept in interventional cardiology [4].

The most common drug coating for DCBs is paclitaxel, which prevents restenosis by stabilizing microtubules and suppressing vascular smooth muscle cell proliferation and migration [5]. Furthermore, following balloon expansion, arterial wall injury triggers an inflammatory response, growth factor release, and smooth muscle cell (SMC) migration. Paclitaxel reduces the release of platelet‐derived growth factors and inhibits the migration of vascular SMCs to the intima [6] [7]. The efficacy of DCBs has previously been established in ISR and small‐vessel disease [8]. Early single‐arm trials demonstrated that side branch treatment using DCBs is safe and associated with favorable angiographic and short‐term clinical outcomes [9] [10] [11]. A meta‐analysis comparing main vessel (MV) stenting with side branch DCB and plain old balloon angioplasty has also shown favorable angiographic and clinical outcomes for DCBs [12].

The hybrid strategy, involving implantation of a DES in the MV and use of a DCB in the side branch, aims to balance the mechanical support of the DES with the antiproliferative properties of the DCB and demonstrates unique advantages. One noteworthy characteristic of the hybrid strategy is the reduction in side branch late lumen loss (SB LLL) observed across multiple studies [10] [12] [13]. However, it should be recognized that LLL is inherently dependent on the initial acute gain achieved during the procedure, and its standalone value as a predictor of success should be interpreted with caution. In this regard, the hybrid strategy appears to offer a favorable “net gain.” More importantly, the potential clinical advantage of this approach may extend beyond surrogate angiographic endpoints to hard clinical outcomes such as major adverse cardiovascular event (MACE) and target lesion revascularization (TLR), although systematic evidence remains limited. By avoiding permanent metal implantation at the side branch ostium, the hybrid strategy not only pharmacologically inhibits intimal hyperplasia but also promotes positive vascular remodeling and effectively restores the vessel′s natural physiological function. Existing clinical data suggest that, in terms of safety, the hybrid strategy is at least noninferior to the DES‐only regimen. A large‐scale analysis of complex coronary lesions showed that DCB‐based strategies, including the hybrid strategy, are associated with a risk of target vessel failure (TVF) comparable to that of DES‐based PCI [14]. Studies such as BIOLUX‐I have also reported favorable safety profiles [9].

Theoretically, the hybrid strategy maintains mechanical support in the MV while using drugs to inhibit intimal hyperplasia at the side branch ostium while preserving the vessel′s original geometry. Although early data are encouraging, there is still a lack of systematic evidence integrating the latest randomized controlled trials (RCTs) and high‐quality observational studies; in particular, the benefits for the high‐risk left main (LM) subgroup remain unclear. Current treatment strategies for bifurcation lesions are being evaluated in several large‐scale, rigorously designed RCTs, and the final results will provide further evidence for the hybrid strategy. These include the PROVISION‐DEB trial [15], the EBC‐DCB trial [16], the SMART‐DCB trial [17], and the RCT designed by Dillen et al. [18], which compare hybrid strategies with provisional or planned double‐stenting. Publication of the results from these RCTs will help fill current evidence gaps and is expected to support an upgrade in the recommendation level of the hybrid strategy in CBLs guidelines.

An updated meta‐analysis of the existing evidence is therefore necessary to evaluate the effects of the hybrid strategy and the stent‐only strategy in the treatment of CBLs, to determine which approach offers better clinical outcomes, and to provide an evidentiary basis for clinical decision‐making.

## 2. Materials and Methods

This study was conducted in strict accordance with the Preferred Reporting Items for Systematic Reviews and Meta‐Analyses (PRISMA 2020) guidelines (Table S1) [19] and was registered with PROSPERO (CRD420251232404). Additional ethical approval was not required, as this study was based on secondary analyses of published RCTs and nonrandomized controlled trials (NRCTs).

### 2.1. Systematic Search

We performed a systematic search of PubMed, Embase, the Cochrane Library, Web of Science, the Chinese National Knowledge Infrastructure (CNKI), and the Wanfang Database from inception to October 19, 2025, without language restrictions. The key search terms were “drug‐coated balloon” and “bifurcation lesion.” Manual searches were also conducted to identify relevant references within the selected articles. The PubMed search strategy was ((“drug‐coated balloon”) OR (“DEB”) OR (“DCB”) OR (“drug‐eluting balloon”)) AND (bifurcation lesion) and was adapted for use in other databases. In addition, reference lists of relevant original and review articles were manually screened. The detailed search strategy for each database, including all MeSH terms, Boolean operators, and specific filters, is provided in Table S2.

### 2.2. Eligibility Criteria

Clinical trials meeting the following criteria were included: (1) RCTs or NRCTs, (2) inclusion of patients with CBLs, (3) comparison of the DES + DCB hybrid strategy versus a DES‐only strategy, and (4) reporting of at least one of the following outcomes during follow‐up: incidence of MACE, TLR, target vessel revascularization (TVR), myocardial infarction (MI), SB LLL, main vessel late lumen loss (MV LLL), or restenosis rate. LLL was defined as the difference between the minimal lumen diameter measured immediately after the procedure and at angiographic follow‐up. Exclusion criteria included isolated ISR lesions, diffuse lesions, single‐arm studies, or studies not reporting outcomes of interest.

### 2.3. Outcome Measures

The primary endpoint was the incidence of MACE. The detailed composite definitions of MACE for each included study are summarized in Table S3. Secondary endpoints included the incidence of TLR, TVR, MI, SB LLL, MV LLL, and restenosis rate.

### 2.4. Data Extraction and Quality Assessment

Two authors (Yuyang Cao and Hao Li) independently conducted database searches, data extraction, and quality assessment based on the above‐mentioned inclusion criteria. Extracted data included (1) first author′s name, publication year, study design, and country; (2) demographic characteristics, including total number of patients, mean age, sex, and coronary heart disease risk factors such as diabetes, hypertension, and hyperlipidemia; (3) lesion characteristics, including Medina classification; and (4) follow‐up duration and outcome measures. Clinical data were extracted for the longest available follow‐up period. Discrepancies were resolved through discussion with a third author (Ying Song).

Risk of bias was assessed using the Cochrane Collaboration′s risk of bias tool [20], which covers seven domains: random sequence generation, allocation concealment, blinding of participants and personnel, blinding of outcome assessors, incomplete outcome data, selective outcome reporting, and other potential sources of bias. Studies were classified as having low, high, or unclear risk of bias. The Newcastle–Ottawa Scale (NOS) [21] was used to assess the quality of NRCTs across three domains: selection of study groups, comparability of groups, and ascertainment of exposure or outcome. The NOS ranges from one to nine stars, with nine stars indicating the highest study quality. In addition to individual study quality assessments, the overall certainty of evidence for each primary and secondary outcome was evaluated using the Grading of Recommendations Assessment, Development, and Evaluation (GRADE) approach. Evidence was classified into four levels of certainty: high, moderate, low, or very low. The assessment considered five specific domains: risk of bias, inconsistency, indirectness, imprecision, and publication bias. A summary of findings (SoF) table (Table S4) was generated using GRADEpro to present the quality of evidence for each clinical and angiographic endpoint.

### 2.5. Statistical Analysis

Continuous variables were analyzed using standardized mean differences (SMDs), and dichotomous variables were analyzed using odds ratios (ORs), both with 95% confidence intervals (CIs). Heterogeneity was assessed using Cochrane′s *Q* test, and the *I* ^2^ statistic was calculated [22]. An *I* ^2^ value greater than 50% [23] was considered indicative of significant heterogeneity, in which case a random‐effects model was applied; otherwise, a fixed‐effects model was used. Subgroup analyses were performed to assess the consistency of results between RCTs and NRCTs. For outcomes with ≥ 10 contributing studies, funnel plots were constructed and visually inspected, and Egger′s regression asymmetry test was performed to evaluate potential small‐study effects [24]. For outcomes with fewer than 10 studies, formal publication‐bias assessment was not performed in accordance with Cochrane Handbook recommendations [22]. Sensitivity analyses were conducted, when necessary, by sequentially excluding individual studies to explore sources of heterogeneity. A *p* value < 0.05 was considered statistically significant. Statistical analyses were performed using RevMan (Version 5.4; Cochrane Collaboration, Oxford, United Kingdom) and StataMP software.

## 3. Results

### 3.1. Database Search and Study Characteristics

**Figure 1:**
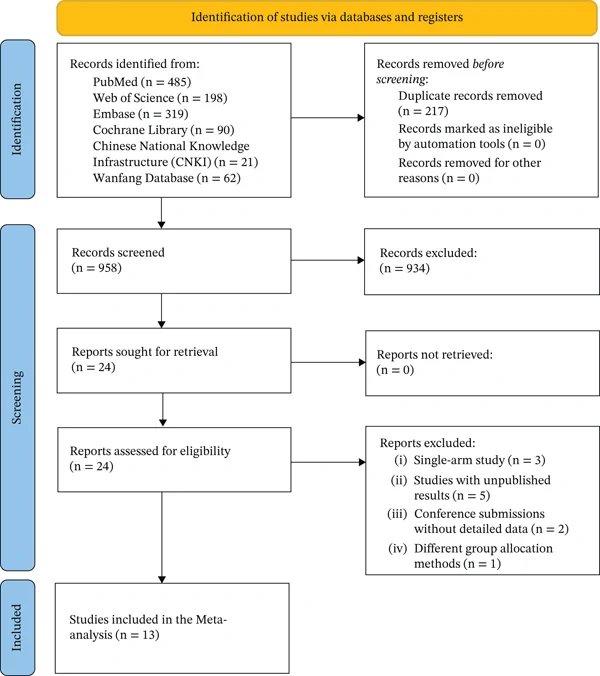
Flowchart of the database search.

The database search process is illustrated in Figure 1. Literature screening and initial baseline characterization identified 1175 records, of which 13 studies were ultimately included [25] [26] [13] [27] [28] [29] [30] [31] [32] [33] [34] [35] [36].

Table 1 summarizes the basic characteristics of the included studies. Overall, five RCTs and eight observational studies were included in the meta‐analysis, comprising a total of 2186 patients (DCB *n* = 1030; DES *n* = 1156). These studies were published between 2019 and 2025 and were conducted in the United Kingdom and China. Sample sizes ranged from 30 to 597 patients, and the mean follow‐up duration ranged from 6 to 20 months. The proportion of male patients ranged from 55% to 81%, and the mean age ranged from 57 to 66 years.

**Table 1:** Patient characteristics for all included studies.

**Author(s)**	**Year**	**Country**	**Study type**	**Treatment (DCB)**	**Treatment (DES)**	**Sample size**	**DCB (n)**	**DES (n)**	**Males (%)**		**Age**	
									**DCB**	**DES**	**DCB**	**DES**
Guo et al.	2023	China	Randomized clinical trial	L‐sandwich and single‐stent + DCB	Double‐stent	99	67	32	53 (79.1)	25 (78.1)	NA	56.78 ± 13.40
Xia et al.	2024	China	Randomized clinical trial	MV‐DES + SB‐DCB	Double‐stent	60	30	30	21 (70)	18 (60)	63.54 ± 5.23	64.37 ± 6.11
Xu et al.	2025	China	Randomized clinical trial	MV‐DES + SB‐DCB	Double‐stent	108	54	54	30 (55.6)	31 (57.4)	58.61 ± 1.37	58.52 ± 1.29
Tang et al.	2025	China	Randomized clinical trial	MV‐DES + SB‐DCB	DES‐only	60	30	30	18 (60)	17 (57)	65.38 ± 3.16	65.57 ± 3.42
Liu	2024	China	Randomized clinical trial	MV‐DES + SB‐DCB	Double‐stent	30	15	15	11 (73.3)	10 (66.7)	60.20 ± 5.29	61.13 ± 4.44
Jones et al.	2023	United Kingdom	Observational	MV‐DES + SB‐DCB	Double‐stent	499	311	188	252 (81.0)	146 (78.0)	61.33 ± 9.4	62.36 ± 11.3
Liu et al.	2022	China	Observational	MV‐DES + SB‐DCB	Double‐stent	100	50	50	38 (76.0)	36 (72.0)	61.76 ± 11.17	64.74 ± 9.20
Pan et al.	2022	China	Observational	MV‐DES + SB‐DCB	DES‐only	597	199	398	145 (72.86)	299 (75.13)	63.75 ± 8.13	64.48 ± 7.75
Huang et al.	2024	China	Observational	MV‐DES + SB‐DCB	Double‐stent	104	48	56	28 (58.3)	32 (57.1)	63.77 ± 9.52	66.14 ± 9.48
Li et al.	2019	China	Observational	MV‐DES + SB‐DCB	Double‐stent	102	44	58	27 (61.4)	33 (56.90)	58.8 ± 10.2	59.2 ± 8.9
Liu et al.	2025	China	Observational	MV‐DES + SB‐DCB	Double‐stent	45	27	18	16 (60.5)	12 (68.2)	61.34 ± 8.78	60.85 ± 7.60
Yang et al.	2024	China	Observational	MV‐DES + SB‐DCB	Double‐stent	216	81	135	51 (63)	87 (64)	61 ± 15	58 ± 12
Cao and Ren	2023	China	Observational	MV‐DES + SB‐DCB	Double‐stent	166	74	92	58 (78.3)	67 (72.8)	61.2 ± 8.3	63.0 ± 7.3
**Author(s)**	**Year**	**Diabetes (%)**		**Hyperlipidemia (%)**		**Hypertension (%)**		**Smoking (%)**		**Medina 1,1,1 (%)**		
		**DCB**	**DES**	**DCB**	**DES**	**DCB**	**DES**	**DCB**	**DES**	**DCB**		**DES**
Guo et al.	2023	22 (32.8)	10 (31.3)	51 (76.1)	20 (62.5)	37 (55.2)	17 (53.1)	31 (46.3)	9 (28.1)	NA		NA
Xia et al.	2024	NA	NA	NA	NA	NA	NA	NA	NA	NA		NA
Xu et al.	2025	13 (24.0)	11 (20.3)	NA	NA	33 (61.1)	34 (63.0)	NA	NA	NA		NA
Tang et al.	2025	NA	NA	NA	NA	NA	NA	NA	NA	NA		NA
Liu	2024	6 (40)	5 (33.3)	NA	NA	8 (53.3)	9 (60)	9 (60)	11 (73.3)	NA		NA
Jones et al.	2023	99 (32.0)	51 (27.1)	211 (68.0)	111 (59.1)	154 (50.0)	101 (54.0)	174 (56.0)	89 (47.4)	199 (64.0)		110 (58.5)
Liu et al.	2022	10 (20.0)	17 (34.0)	2 (4.0)	5 (10.0)	26 (52.0)	18 (36.0)	25 (50.0)	20 (40.0)	44 (88.0)		41 (82.0)
Pan et al.	2022	77 (38.69)	165 (41.46)	54 (27.14)	119 (29.90)	107 (53.77)	205 (51.51)	72 (36.18)	138 (34.67)	167 (83.92)		316 (79.40)
Huang et al.	2024	24 (50)	26 (46.4)	NA	NA	32 (66.7)	34 (60.7)	29 (60.4)	28 (50)	13 (27.1)		15 (26.8)
Li et al.	2019	12 (27.27)	13 (22.41)	25 (56.81)	34 (58.62)	13 (29.55)	16 (27.59)	21 (47.73)	30 (51.72)	NA		NA
Liu et al.	2025	15 (55.4)	11 (60.4)	NA	NA	17 (64.2)	11 (61.2)	16 (58.1)	10 (56.1)	27 (100)		18 (100)
Yang et al.	2024	24 (30)	37 (27)	21 (26)	36 (27)	46 (57)	81 (60)	44 (54)	78 (58)	25 (31)		26 (19)
Cao and Ren	2023	35 (47.2)	31 (33.6)	NA	NA	46 (62.1)	40 (43.4)	46 (62.1)	54 (58.6)	NA		NA

### 3.2. Risk of Bias Assessment

**Figure 2:**
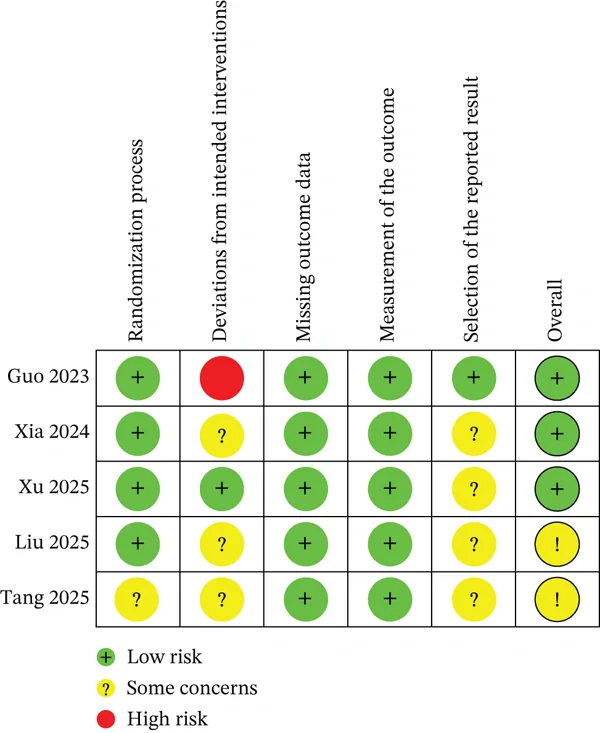
Quality evaluation of the included RCTs.

**Figure 3:**
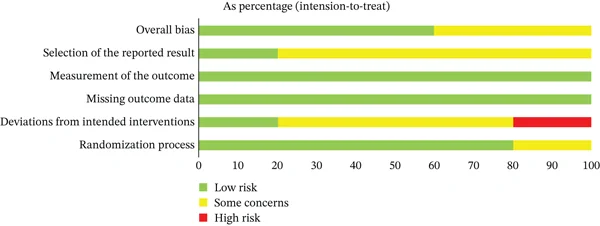
Quality evaluation of the included RCTs as a percentage.

Among the five included RCTs, three were rated as low risk in the RoB assessment, whereas two were rated as having some concerns. Owing to the nature of the intervention, none of the trials used blinding, resulting in an overall quality assessment as shown in Figures 2 and 3. The quality of the included observational studies was generally high, with NOS scores ranging from eight to nine stars (Table 2).

**Table 2:** Quality evaluation of the included NRCT.

Study	Representativeness of the exposed cohort	Selection of the nonexposed cohort	Ascertainment of the exposure	Demonstration that the outcome of interest was not present at the start of the study	Comparability—Age and gender	Comparability—Other factors	Assessment of outcome	Was the follow‐up long enough for outcomes to occur	Adequacy of follow‐up of cohorts	Total
Jones et al. (2023)	1	1	1	1	1	1	1	1	1	9
Liu et al. (2022)	0	1	1	1	1	1	1	1	1	8
Pan et al. (2022)	0	1	1	1	1	1	1	1	1	8
Huang et al. (2024)	0	1	1	1	1	1	1	1	1	8
Li et al. (2019)	0	1	1	1	1	1	1	1	1	8
Liu et al. (2025)	1	1	1	1	1	1	1	1	1	9
Yang et al. (2024)	1	1	1	1	1	1	1	1	1	9
Cao and Ren (2023)	1	1	1	1	1	1	1	1	1	9

### 3.3. Quality of Evidence (GRADE Assessment)

The overall certainty of evidence for the primary outcome (MACE) was rated as moderate, with the primary reason for downgrading being the inclusion of nonrandomized observational studies. For the secondary outcomes, the certainty of evidence for MI, side branch restenosis, and TLR was also rated as moderate, whereas TVR was rated as low due to imprecision. The certainty for SB LLL was rated as very low due to extreme statistical heterogeneity. Detailed GRADE assessments are summarized in Table S4.

### 3.4. Meta‐Analysis Results

#### 3.4.1. Primary Outcome

**Figure 4:**
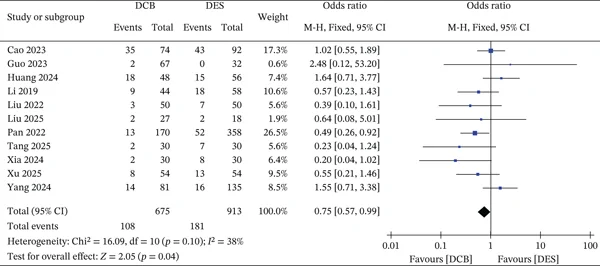
Forest plot for MACE in the hybrid strategy versus the DES‐only strategy.

Eleven studies reported the incidence of MACE in the hybrid strategy group (*n* = 675) and the DES‐only group (*n* = 913). The incidence of MACE was significantly lower in the hybrid strategy group than in the DES‐only group (OR 0.75; 95% CI 0.57–0.99; *p* = 0.04; *I* ^2^ = 38*%*) (Figure 4).

**Figure 5:**
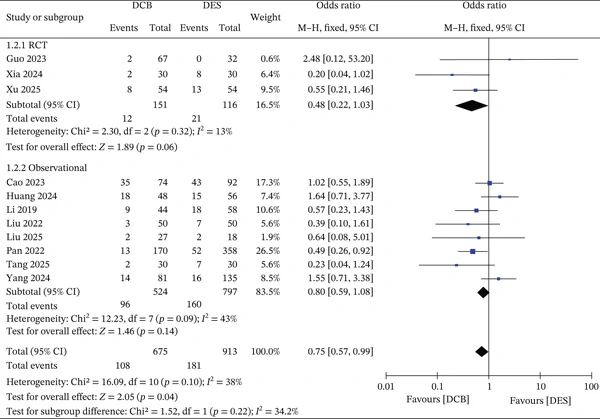
Forest plot for subgroup analysis of MACE in the hybrid strategy versus the DES‐only strategy by RCTs or observational studies.

Subgroup analysis showed that the ORs were not statistically significant when RCTs or observational studies were analyzed separately (*p* = 0.06 and *p* = 0.14, respectively), likely owing to limited sample sizes or heterogeneity (Figure 5).

**Figure 6:**
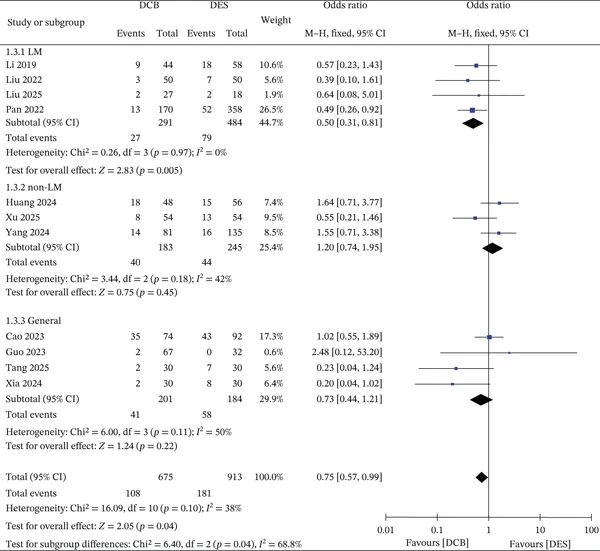
Forest plot for subgroup analysis of MACE in the hybrid strategy versus the DES‐only strategy by lesion location.

However, subgroup analysis by lesion location—LM, non‐LM, and overall (including both LM and non‐LM lesions)—demonstrated that, among patients with LM lesions, the hybrid strategy was associated with a significantly lower risk of MACE compared with DES alone (OR 0.50; 95% CI 0.31 to 0.81; *p* = 0.005; *I* ^2^ = 0*%*). Lesion location may therefore represent the primary source of heterogeneity (*I* ^2^ = 38*%*) in the overall analysis (Figure 6).

As a supplementary analysis, we also compared the hybrid strategy directly with the double‐stent subgroup alone (nine studies). No statistically significant difference was observed (OR 0.89; 95% CI 0.65–1.23; *p* = 0.48; Figure S3). This result should be interpreted with caution, as the double‐stent group was heterogeneously defined across studies, and several studies did not separately report outcomes by control‐arm stenting approach.

#### 3.4.2. Secondary Outcomes

##### 3.4.2.1. TLR

**Figure 7:**
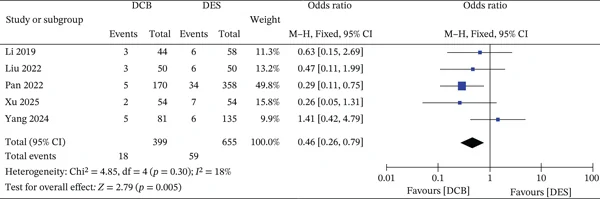
Forest plot for TLR in the hybrid strategy versus the DES‐only strategy.

Five studies compared differences in TLR (DCB *n* = 399; DES *n* = 655). Compared with the DES‐only group, the hybrid strategy was associated with a significantly lower risk of TLR (OR 0.46; 95% CI 0.26–0.79; *p* = 0.005; *I* ^2^ = 18*%*), with low heterogeneity across studies (Figure 7).

##### 3.4.2.2. Late Lumen Loss (Side Branch)

**Figure 8:**
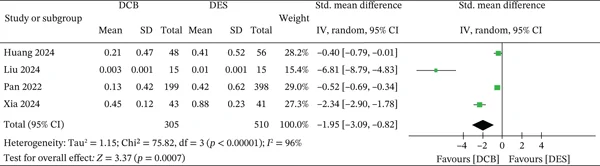
Forest plot for late lumen loss of the side branch in the hybrid strategy versus the DES‐only strategy.

Four studies compared SB LLL (DCB *n* = 305; DES *n* = 510). All four studies reported numerically lower SB LLL with the hybrid strategy. However, extreme statistical heterogeneity was present (*I* ^2^ = 96*%*, Figure 8), rendering the pooled estimate (SMD −1.95; 95% CI −3.09 to −0.82) essentially uninterpretable as a single summary measure. This heterogeneity likely reflects differences in DCB type (paclitaxel vs. sirolimus), lesion preparation, angiographic measurement protocols, and follow‐up durations. Given the “very low” GRADE certainty, this result should be interpreted as directionally consistent but nonpoolable.

##### 3.4.2.3. Late Lumen Loss (Main Vessel)

**Figure 9:**
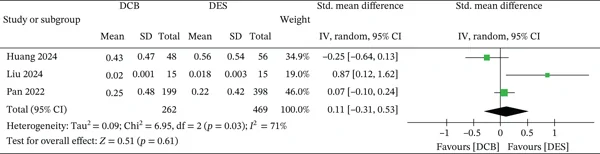
Forest plot for late lumen loss of the main vessel in the hybrid strategy versus the DES‐only strategy.

Three studies compared MV LLL (DCB *n* = 262; DES *n* = 469). No statistically significant difference was observed between the two groups (*p* = 0.61) (Figure 9).

##### 3.4.2.4. Restenosis (Side Branch)

**Figure 10:**
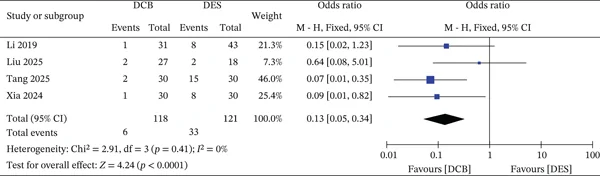
Forest plot for restenosis of the side branch in the hybrid strategy versus the DES‐only strategy.

Four studies compared the incidence of side branch lumen restenosis (DCB *n* = 118; DES *n* = 121). Compared with the DES‐only strategy, the hybrid strategy significantly reduced the risk of lumen restenosis (OR 0.13; 95% CI 0.05–0.34; *p* < 0.0001; *I* ^2^ = 0*%*) (Figure 10).

##### 3.4.2.5. MI

**Figure 11:**
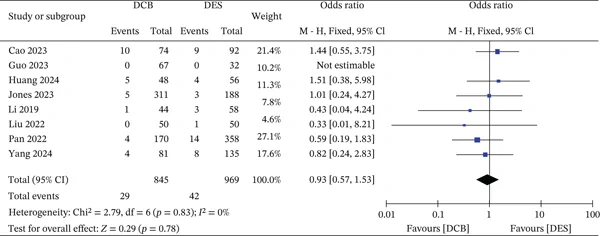
Forest plot for myocardial infarction in the hybrid strategy versus the DES‐only strategy.

Eight studies compared the incidence of MI (DCB *n* = 845; DES *n* = 969). No statistically significant difference in MI risk was observed between the hybrid and DES‐only strategies (OR 0.93; 95% CI 0.57–1.53; *p* = 0.78; *I* ^2^ = 0*%*). These findings indicate that the hybrid strategy has a safety profile comparable to that of the DES‐only strategy and does not increase the risk of perioperative or late MI (Figure 11).

##### 3.4.2.6. TVR

**Figure 12:**
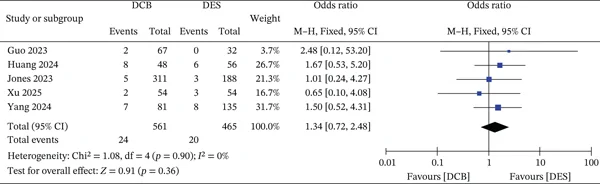
Forest plot for TVR in the hybrid strategy versus the DES‐only strategy.

Five studies compared TVR (DCB *n* = 561; DES *n* = 465). No statistically significant difference in TVR risk was observed between the hybrid and DES‐only strategies (OR 1.34; 95% CI 0.72–2.48; *p* = 0.36). This suggests that, for the treatment of CBLs, there was no significant difference between the hybrid strategy and the traditional stent‐only strategy with respect to long‐term revascularization (Figure 12).

#### 3.4.3. LM Subgroup Analysis

**Figure 13:**
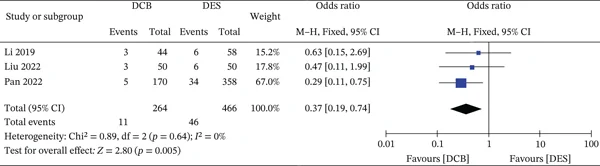
Forest plot for TLR of the left main in the hybrid strategy versus the DES‐only strategy.

In the LM subgroup, three studies compared TLR (DCB *n* = 264; DES *n* = 466). For LM lesions, the hybrid strategy significantly reduced the risk of TLR compared with the DES‐only strategy (OR 0.37; 95% CI 0.19–0.74; *p* = 0.005; *I* ^2^ = 0*%*) (Figure 13).

**Figure 14:**
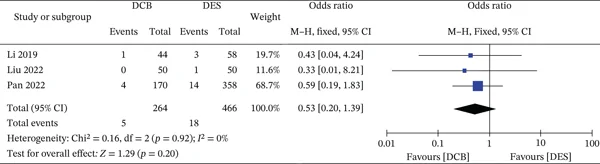
Forest plot for MI of the left main in the hybrid strategy versus the DES‐only strategy.

Regarding MI incidence in this subgroup, no statistically significant difference was observed between the hybrid and DES‐only strategies (*p* = 0.20). This indicates that, in LM lesions, the hybrid strategy has a safety profile comparable to that of the DES‐only strategy and does not increase the risk of perioperative or late MI, consistent with the overall analysis (Figure 14).

#### 3.4.4. Publication Bias

Among all outcomes, only MACE was assessed by ≥ 10 contributing studies (*k* = 11). Visual inspection of the funnel plot for MACE (Figure S1) did not reveal marked asymmetry, and Egger′s regression test showed no statistically significant evidence of small‐study effects (Figure S2). For the remaining outcomes (TLR, TVR, SB LLL, MV LLL, MI, and restenosis), formal assessment of publication bias was not performed, as funnel‐plot inspection and Egger′s test are not recommended when fewer than 10 studies contribute to an analysis (Cochrane Handbook [22]). Funnel plots for these outcomes are retained in the Supporting Information for visual reference, but their results should not be interpreted as evidence of low publication‐bias risk.

## 4. Discussion

This meta‐analysis aimed to systematically evaluate the efficacy and safety of a hybrid strategy combining DCBs and DES compared with a DES‐only strategy for the treatment of CBLs. Our primary finding is that the hybrid strategy not only significantly reduced the risks of MACE and TLR but also markedly reduced side branch restenosis at the anatomical level without increasing the risk of perioperative MI.

Compared with the DES‐only strategy, patients treated with the hybrid approach experienced a significantly lower incidence of MACE. With respect to lesion‐related endpoints, the hybrid strategy significantly reduced the risk of TLR, and for anatomical endpoints, it significantly lowered the incidence of side branch restenosis. These findings suggest that the observed reduction in MACE may be driven by fewer recurrent events at the local lesion site. Application of DCBs in the side branch effectively addresses one of the most common post‐treatment complications of bifurcation lesions—side branch ostial restenosis. The uniformly low risk of side branch restenosis provides strong anatomical evidence that the local therapeutic effect of the DCB strategy in bifurcation side branches is reliable and consistent.

The efficacy of the hybrid strategy is not incidental but is grounded in the unique drug delivery mechanism of DCBs and their protective effect on bifurcation anatomy. A key advantage of DCBs is their ability to provide local, transient drug delivery while avoiding permanent implantation of metal stents or durable polymers at the side branch ostium [4]. In traditional double‐stent techniques, additional stent implantation in the side branch increases the risks of stent overlap, ISR, and late thrombosis, which are closely associated with chronic inflammation and delayed endothelial healing caused by permanent polymers [37]. In contrast, DCBs promote more physiological vascular healing by delivering antiproliferative drugs without leaving a permanent implant. This feature is particularly advantageous for patients at HBR, who may benefit from shorter durations of dual antiplatelet therapy [3].

During PCI for bifurcation lesions, balloon dilation of the side branch ostium after MV stenting—regardless of whether a DCB is used—inevitably induces some degree of arterial wall injury, triggering inflammatory responses and growth factor release [37]. The local, high‐concentration release of paclitaxel effectively reduces the release of platelet‐derived growth factors and potently inhibits subsequent SMC proliferation [38]. This localized, high‐efficiency antiproliferative drug delivery provides the pharmacological basis for the significant reductions in TLR and side branch restenosis. Importantly, the DCB strategy pharmacologically suppresses excessive neointimal hyperplasia while maximally preserving native vessel geometry and the integrity of the carina [39]. Among the included studies, the specific DCB drug type was not uniformly reported. Notably, one large observational study (*n* = 499) explicitly used a sirolimus‐eluting balloon for side‐branch treatment [26], only two studies specified the use of paclitaxel‐coated balloons, and the remaining studies did not report the DCB drug or brand. Sirolimus exerts its antiproliferative effect through a distinct mechanism: It binds to the intracellular receptor FKBP12 to form a complex that inhibits the mammalian target of rapamycin (mTOR), thereby arresting the cell cycle at the G1‐to‐S phase transition and suppressing SMC proliferation and migration [40]. Compared with the cytotoxic mechanism of paclitaxel (microtubule stabilization leading to cell death) [38], the cytostatic action of sirolimus may offer a broader therapeutic window and a more favorable vascular healing profile [41]. However, the inclusion of pharmacologically heterogeneous DCB types represents a source of heterogeneity that may partly explain the extreme variability observed in the SB LLL analysis (*I* ^2^ = 96*%*). The recently reported SPACIOUS trial demonstrated noninferiority of sirolimus‐coated balloons compared with paclitaxel‐coated balloons for side‐branch diameter stenosis in non‐LM bifurcation lesions [42], suggesting comparable efficacy of both drug types. Nonetheless, further studies are needed to confirm whether the two drug coatings yield equivalent long‐term clinical outcomes in this setting.

Beyond the question of which specific drug coating is optimal, a more fundamental interpretive consideration arises regarding the mechanism underlying the overall benefit of the hybrid strategy. It should be noted that the observed clinical benefits of the hybrid strategy reflect both a structural advantage—avoiding a second permanent stent in the side branch—and a pharmacological advantage provided by the DCB. In 11 of the 13 included studies, the control arm employed a double‐stent technique; therefore, the pooled comparison predominantly reflects “MV stenting with SB‐DCB” versus “planned double stenting.” The well‐established disadvantages of routine double‐stenting—including increased procedural complexity, stent overlap, and chronic inflammation—likely contribute to the relative superiority of the hybrid approach. When the hybrid strategy was directly compared with the double‐stent subgroup alone in an exploratory analysis, no significant difference was observed (OR 0.89; 95% CI 0.65–1.23; *p* = 0.48; Figure S3), suggesting that the magnitude of incremental benefit may be modest when the comparator is restricted to double‐stenting. To definitively isolate the independent pharmacological contribution of DCBs from the structural benefit of stent avoidance, future trials comparing the hybrid strategy with provisional stenting using a plain balloon (POBA) for the side branch would be required.

The clinical benefits of the hybrid strategy are localized to the bifurcation region, specifically the side branch ostium, where the DCB delivers the antiproliferative drug effectively. The antiproliferative effect of DCBs prevents excessive intimal hyperplasia at the side branch ostium, thereby reducing the risk of TLR. However, this localized therapy does not influence atherosclerotic progression or restenosis risk in distal segments of the MV or in nonindex lesion areas within the target vessel. The nonsignificant TVR results therefore support the conclusion that the therapeutic effect of DCBs is precise and lesion‐specific rather than a global intervention for the entire target vessel.

Although the direction of effect for SB LLL was consistent across studies, the results could not be pooled quantitatively because of substantial heterogeneity. This variability likely arises from technical and methodological differences across studies. LLL is an angiographic anatomical endpoint, and in complex three‐dimensional bifurcation anatomy, two‐dimensional angiography has inherent limitations in accurately assessing the side branch ostial lumen, as measurements are sensitive to projection angles and the timing of angiography. Following MV stenting, side branch geometry becomes more complex, further increasing subjectivity and variability in LLL measurement. The observed heterogeneity may also be partly attributable to differences in study design. In particular, DCB drug type varied across studies—as noted above, Jones et al. [26] used a sirolimus‐coated balloon while most studies did not specify the drug type—introducing potential pharmacological heterogeneity into the pooled analysis. Additional sources of variability include lesion preparation techniques (e.g., noncompliant balloons, cutting balloons, or rotational atherectomy [43]) and postoptimization strategies such as proximal optimization technique and kissing balloon inflation [44].

LM‐CBLs, owing to their anatomical importance and high‐risk nature, remain among the most challenging scenarios in interventional cardiology. Our analysis indicated a potential benefit of the hybrid strategy in reducing MACE. However, it is important to emphasize that this LM subgroup analysis was based on a limited number of studies (*n* = 3). Consequently, these findings should be interpreted with caution and considered strictly exploratory and hypothesis‐generating. The current preliminary evidence suggests a promising direction, but large‐scale, dedicated RCTs are required to definitively confirm the efficacy and safety of the hybrid approach in complex LM lesions. The absence of heterogeneity in the LM subgroup suggests that the hybrid strategy provides stable and consistent therapeutic benefits in this anatomically demanding population. Compared with complex double‐stent techniques, such as the DK‐Crush strategy [45], the hybrid approach simplifies procedural workflow and reduces long‐term risks associated with multilayer stent overlap, stent deformation, and underexpansion. By avoiding additional metal implantation, DCB use facilitates restoration of hemodynamic integrity at the LM bifurcation without increasing metal burden or chronic inflammation, which may explain the consistent and favorable clinical outcomes observed in this subgroup.

Eight studies reported MI incidence and demonstrated no significant difference in MI risk between the hybrid and stent‐only strategies, with zero heterogeneity. These findings were consistent in the LM subgroup. In provisional stenting strategies, balloon dilation of the side branch after MV stenting may raise concerns regarding an increased risk of perioperative side branch occlusion or MI owing to procedural manipulation, such as guidewire recrossing or balloon dilation. However, our meta‐analysis indicates that side branch treatment with DCB does not increase the risk of acute or subacute adverse events.

Finally, this study has several methodological limitations. Regarding the comparison between the hybrid strategy and a strictly single‐stent strategy, we encountered a limitation in the available literature. Two of our included studies did not differentiate between single and double stenting in their control groups, reporting them instead as a mixed “DES‐only” cohort, which prevented a robust, isolated meta‐analysis exclusively against a single‐stent strategy. Additionally, the clinical outcomes were based on the endpoint definitions provided by each individual study, which contributes to clinical heterogeneity. Specifically, varying definitions of MACE across the included studies may have introduced reporting bias. As detailed in Table S3, the components of MACE ranged widely from strict ischemic events (e.g., cardiac death, MI, and TVR) to broader clinical presentations, including angina, heart failure, and arrhythmia. Furthermore, it is noteworthy that all 13 included studies were conducted in the United Kingdom and China. This geographic concentration limits the generalizability and external validity of our conclusions. Future validation in more diverse, global populations is explicitly needed to confirm these findings across different demographics. Furthermore, design‐stratified subgroup analyses for secondary endpoints were not performed because the limited number of contributing studies would yield subgroups too small for meaningful interpretation.

Additionally, the pooled estimates were influenced to a considerable extent by two large observational studies (Jones et al., *n* = 499; Pan et al., *n* = 597), which together contributed the majority of the total sample size. Because observational studies are inherently susceptible to confounding by indication—for instance, operators may preferentially select the hybrid strategy for anatomically less complex side branches—the pooled effect should be interpreted with this caveat in mind. Notably, the RCT subgroup approached but did not reach statistical significance for MACE (*p* = 0.06), which is consistent with the more limited sample sizes of individual trials rather than necessarily indicating an absence of effect. Nonetheless, this borderline primary result—not reproduced within either design subgroup (RCTs *p* = 0.06; observational studies *p* = 0.14)—should be interpreted with caution. These considerations reinforce the importance of future adequately powered, multicenter RCTs to provide more definitive evidence regarding the hybrid strategy. Furthermore, while our meta‐analysis primarily focused on the angiographic and procedural differences between the hybrid and stent‐only strategies, it is crucial to recognize that long‐term clinical outcomes (e.g., MACE) are deeply influenced by patients′ underlying metabolic profiles. Recent evidence strongly highlights that baseline systemic metabolic burdens—such as longitudinal variations in triglyceride–glucose indices [46] and admission glycemic variability [47]—are powerful independent predictors of adverse cardiovascular events following acute coronary syndromes and complex interventions. However, the primary studies included in our analysis largely lacked granular data on these specific metabolic or glycemic parameters, precluding a more stratified analysis. Future randomized trials evaluating bifurcation strategies should explicitly incorporate these baseline metabolic risk modifiers to better identify which patient subgroups derive the most substantial long‐term benefit from the hybrid approach.

## 5. Conclusion

This meta‐analysis suggests that, in the interventional treatment of CBLs, a hybrid strategy combining DCBs with DES may reduce the risks of MACE and TLR compared with a stent‐only strategy. These benefits are likely attributable to the dual advantages of the hybrid approach: avoidance of a second permanent metallic implant in the side branch—thereby reducing stent‐related complications such as chronic inflammation, stent overlap, and delayed healing—and the localized antiproliferative drug delivery by the DCB, which suppresses neointimal hyperplasia at the side branch ostium. Although the predominance of double‐stent controls in the included studies means that the relative magnitude of each mechanism cannot be individually quantified, the consistent direction of benefit across clinical and angiographic outcomes supports the hybrid approach as a viable and potentially optimized alternative to complex double‐stent techniques for bifurcation lesions.

## Author Contributions

Yuyang Cao and Hao Li contributed equally and were joint first authors.

## Funding

This study was funded by the Capital Health Development Research Project (2026‐2‐4036), the National High Level Hospital Clinical Research Funding (2024‐GSP‐TJ‐8), the CAMS Innovation Fund for Medical Sciences (2023‐I2M‐1‐002 and 2023‐I2M‐C&T‐B‐061), and the Special Project of “Science and Technology Deputy Director” in Changping District (T2025‐ZX057).

## Conflicts of Interest

The authors declare no conflicts of interest.

## Supporting information


**Supporting Information** Additional supporting information can be found online in the Supporting Information section. Figure S1: Funnel plots for the publication biases underlying the meta‐analyses regarding MACE, TLR, LLL of SB, LLL of MV, restenosis, MI, TVR, TLR of LM, and MI of LM. Figure S2: Egger′s test for MACE in hybrid strategy versus DES‐only strategy. Figure S3: Forest plot comparing the hybrid strategy with the double‐stent subgroup alone for major adverse cardiovascular events (MACE). Table S1: PRISMA 2020 Checklist. Table S2: Search strategies for each database (search date: October 19, 2025). Table S3: Definitions of major adverse cardiovascular events (MACE) and follow‐up duration across included studies. Table S4: GRADE summary of findings table.

## Data Availability

Data sharing is not applicable to this article as no datasets were generated or analyzed during the current study.
