# Supplementary material for: Comparison of Hybrid Strategy With Drug‐Coated Balloons Versus Stent‐Only Strategy for Coronary Bifurcation Lesions: A Systematic Review and Meta‐Analysis
Source: Cardiovasc Ther. 2026 Sep 24;2026:7598895. doi: 10.1155/cdr/7598895 (PMC13602816; doi:10.1155/cdr/7598895)
Supplement: Supplementary file 1 [file CDR-2026-7598895-s001.docx]

# Supplementary Material

**Figure S1.** Funnel plots for the publication biases underlying the meta-analyses. (a) MACE. (b) TLR. (c)LLL of SB. (d)LLL of MV. (e)Restenosis. (f)MI. (g)TVR. (h)TLR of LM. (k)MI of LM.

**Figure S2**. Egger’s test for MACE in Hybrid Strategy vs DES-only Stratege.

**Figure S3**. Forest plot comparing the hybrid strategy with the double-stent subgroup alone for major adverse cardiovascular events (MACE).

**Table S1**. PRISMA 2020 Checklist

| **Section and Topic** | **Item #** | **Checklist item** | **Location where item is reported** |
| --- | --- | --- | --- |
| **TITLE** | | |  |
| Title | 1 | Identify the report as a systematic review. | Page 1 |
| **ABSTRACT** | | |  |
| Abstract | 2 | See the PRISMA 2020 for Abstracts checklist. | Page 1 |
| **INTRODUCTION** | | |  |
| Rationale | 3 | Describe the rationale for the review in the context of existing knowledge. | Page 3 |
| Objectives | 4 | Provide an explicit statement of the objective(s) or question(s) the review addresses. | Page 5 |
| **METHODS** | | |  |
| Eligibility criteria | 5 | Specify the inclusion and exclusion criteria for the review and how studies were grouped for the syntheses. | Page 5 |
| Information sources | 6 | Specify all databases, registers, websites, organisations, reference lists and other sources searched or consulted to identify studies. Specify the date when each source was last searched or consulted. | Page 5 |
| Search strategy | 7 | Present the full search strategies for all databases, registers and websites, including any filters and limits used. | Page 5 |
| Selection process | 8 | Specify the methods used to decide whether a study met the inclusion criteria of the review, including how many reviewers screened each record and each report retrieved, whether they worked independently, and if applicable, details of automation tools used in the process. | Page 6 |
| Data collection process | 9 | Specify the methods used to collect data from reports, including how many reviewers collected data from each report, whether they worked independently, any processes for obtaining or confirming data from study investigators, and if applicable, details of automation tools used in the process. | Page 6 |
| Data items | 10a | List and define all outcomes for which data were sought. Specify whether all results that were compatible with each outcome domain in each study were sought (e.g. for all measures, time points, analyses), and if not, the methods used to decide which results to collect. | Page 6 |
|  | 10b | List and define all other variables for which data were sought (e.g. participant and intervention characteristics, funding sources). Describe any assumptions made about any missing or unclear information. | N/A |
| Study risk of bias assessment | 11 | Specify the methods used to assess risk of bias in the included studies, including details of the tool(s) used, how many reviewers assessed each study and whether they worked independently, and if applicable, details of automation tools used in the process. | Page 6 |
| Effect measures | 12 | Specify for each outcome the effect measure(s) (e.g. risk ratio, mean difference) used in the synthesis or presentation of results. | Page 7 |
| Synthesis methods | 13a | Describe the processes used to decide which studies were eligible for each synthesis (e.g. tabulating the study intervention characteristics and comparing against the planned groups for each synthesis (item #5)). | Page 9, Table 1 |
|  | 13b | Describe any methods required to prepare the data for presentation or synthesis, such as handling of missing summary statistics, or data conversions. | Page 7 |
|  | 13c | Describe any methods used to tabulate or visually display results of individual studies and syntheses. | Page 7, Page 8 |
|  | 13d | Describe any methods used to synthesize results and provide a rationale for the choice(s). If meta-analysis was performed, describe the model(s), method(s) to identify the presence and extent of statistical heterogeneity, and software package(s) used. | Page 7, Page 8 |
|  | 13e | Describe any methods used to explore possible causes of heterogeneity among study results (e.g. subgroup analysis, meta-regression). | Page 7, Page 8 |
|  | 13f | Describe any sensitivity analyses conducted to assess robustness of the synthesized results. | Page 7, Page 8 |
| Reporting bias assessment | 14 | Describe any methods used to assess risk of bias due to missing results in a synthesis (arising from reporting biases). | Page 7 |
| Certainty assessment | 15 | Describe any methods used to assess certainty (or confidence) in the body of evidence for an outcome. | Page 7 |
| **RESULTS** | | |  |
| Study selection | 16a | Describe the results of the search and selection process, from the number of records identified in the search to the number of studies included in the review, ideally using a flow diagram. | Page 8, Figure 1 |
|  | 16b | Cite studies that might appear to meet the inclusion criteria, but which were excluded, and explain why they were excluded. | N/A |
| Study characteristics | 17 | Cite each included study and present its characteristics. | Page8, Table 1 |
| Risk of bias in studies | 18 | Present assessments of risk of bias for each included study. | Page 11, Figure 2, Figure 3,Table 2 |
| Results of individual studies | 19 | For all outcomes, present, for each study: (a) summary statistics for each group (where appropriate) and (b) an effect estimate and its precision (e.g. confidence/credible interval), ideally using structured tables or plots. | Page9, Page 10,Table1 |
| Results of syntheses | 20a | For each synthesis, briefly summarise the characteristics and risk of bias among contributing studies. | Page 11 |
|  | 20b | Present results of all statistical syntheses conducted. If meta-analysis was done, present for each the summary estimate and its precision (e.g. confidence/credible interval) and measures of statistical heterogeneity. If comparing groups, describe the direction of the effect. | Page 14, Page 15 Page 16 |
|  | 20c | Present results of all investigations of possible causes of heterogeneity among study results. | Page 14, Page 15 Page 16 |
|  | 20d | Present results of all sensitivity analyses conducted to assess the robustness of the synthesized results. | Page 14, Page 15 Page 16 |
| Reporting biases | 21 | Present assessments of risk of bias due to missing results (arising from reporting biases) for each synthesis assessed. | Page 14, Page 15 Page 16 |
| Certainty of evidence | 22 | Present assessments of certainty (or confidence) in the body of evidence for each outcome assessed. | Page 14, Page 15 Page 16 |
| **DISCUSSION** | | |  |
| Discussion | 23a | Provide a general interpretation of the results in the context of other evidence. | Page 16, Page 17, Page 18, Page 19, Page 20 |
|  | 23b | Discuss any limitations of the evidence included in the review. | Page 20 |
|  | 23c | Discuss any limitations of the review processes used. | Page 19, Page 20 |
|  | 23d | Discuss implications of the results for practice, policy, and future research. | Page 19, Page 20 |
| **OTHER INFORMATION** | | |  |
| Registration and protocol | 24a | Provide registration information for the review, including register name and registration number, or state that the review was not registered. | Page 5 |
|  | 24b | Indicate where the review protocol can be accessed, or state that a protocol was not prepared. | N/A |
|  | 24c | Describe and explain any amendments to information provided at registration or in the protocol. | N/A |
| Support | 25 | Describe sources of financial or non-financial support for the review, and the role of the funders or sponsors in the review. | declarationStatement |
| Competing interests | 26 | Declare any competing interests of review authors. | declarationStatement |
| Availability of data, code and other materials | 27 | Report which of the following are publicly available and where they can be found: template data collection forms; data extracted from included studies; data used for all analyses; analytic code; any other materials used in the review. | N/A |

Table S2. Search strategies for each database (Search Date: October 19, 2025)

| Database | Search Strategy |
| --- | --- |
| PubMed | (("drug-coated balloon") OR ("DEB") OR ("DCB") OR ("drug-eluting balloon")) AND (bifurcation lesion) |
| Embase | ('drug-eluting balloon'/exp OR 'drug coated balloon' OR 'dcb' OR 'deb') AND ('artery bifurcation'/exp OR 'bifurcation lesion') |
| The Cochrane Library | (("drug-coated balloon") OR ("DEB") OR ("DCB") OR ("drug-eluting balloon")) AND (bifurcation lesion) |
| Web of Science | (("drug-coated balloon") OR ("DEB") OR ("DCB") OR ("drug-eluting balloon")) AND (bifurcation lesion) |
| CNKI (知网) | (主题:药物球囊 + 药物球囊扩张术 + 药物球囊技术) AND (主题:分叉病变 + 冠脉分叉病变) |
| Wanfang (万方) | 主题:(药物球囊 + 药物球囊扩张术 + 药物球囊技术) AND 主题:(分叉病变 + 冠脉分叉病变) |

Table S3. Definitions of Major Adverse Cardiovascular Events (MACE) and Follow-up Duration Across Included Studies

| **Study (Author, Year)** | **Country** | **Follow-up** | **Definition of MACE (Composite Endpoints)** |
| --- | --- | --- | --- |
| **Cao et al., 2023** | China | 20 months | Angina, myocardial infarction (MI), and all-cause mortality. |
| **Guo et al., 2023** | China | 6 months | Acute MI, cardiac death, and target vessel revascularization (TVR). |
| **Huang et al., 2024** | China | 9 months | Cardiac death, TVR, acute MI, and malignant arrhythmia. |
| **Jones et al., 2023** | UK | Not specified* | All-cause mortality, TVR, and target vessel myocardial infarction (TV-MI). |
| **Li et al., 2019** | China | 12 months | Angina, MI, cardiac death, in-stent restenosis (ISR), target lesion revascularization (TLR), and stent thrombosis. |
| **Liu et al., 2022** | China | Not specified* | TLR, MI, and cardiac death. |
| **Liu et al., 2024** | China | 6 months | Not explicitly detailed (0 MACE events reported during the follow-up period). |
| **Liu et al., 2025** | China | 12 months | Angina, stent thrombosis, ISR, MI, TLR, and cardiac death. |
| **Pan et al., 2022** | China | Not specified* | Heart failure, arrhythmia, cardiac death, and MI. |
| **Tang et al., 2025** | China | Not specified* | Heart failure, arrhythmia, cardiac death, and MI. |
| **Xia et al., 2024** | China | 12 months | MI, heart failure, and stroke. |
| **Xu et al., 2025** | China | 9 months | Recurrent MI, recurrent angina, all-cause mortality, and TVR. |
| **Yang et al., 2024** | China | 12 months | Recurrent angina, cardiac death, recurrent MI, and TVR. |

Table S4: GRADE Summary of Findings Table

**Question:** Hybrid Strategy compared to DES-only for bifurcation lesions

| **Certainty assessment** | | | | | | | **№ of patients** | | **Effect** | | **Certainty** | **Importance** |
| --- | --- | --- | --- | --- | --- | --- | --- | --- | --- | --- | --- | --- |
| **№ of studies** | **Study design** | **Risk of bias** | **Inconsistency** | **Indirectness** | **Imprecision** | **Other considerations** | **Hybrid Strategy** | **DES-only** | **Relative (95% CI)** | **Absolute (95% CI)** |  |  |
| **MACE** | | | | | | | | | | | | |
| 11 | randomised trials and observational studies | serious^a^ | not serious | not serious | not serious | none | 108/675 (16.0%) | 181/913 (19.8%) | **OR 0.75** (0.57 to 0.99) | **42 fewer per 1,000** (from 75 fewer to 2 fewer) | ⨁⨁⨁◯ Moderate^a^ | CRITICAL |
| **TLR** | | | | | | | | | | | | |
| 5 | randomised trials and observational studies | serious^a^ | not serious | not serious | not serious | none | 18/399 (4.5%) | 59/655 (9.0%) | **OR 0.46** (0.26 to 0.79) | **47 fewer per 1,000** (from 65 fewer to 18 fewer) | ⨁⨁⨁◯ Moderate^a^ | CRITICAL |
| **LLL（SB）** | | | | | | | | | | | | |
| 4 | randomised trials and observational studies | serious^a^ | very serious^b^ | not serious | not serious | none | 305 | 510 | - | SMD **1.95 lower** (3.09 lower to 0.82 lower) | ⨁◯◯◯ Very low^a,b^ | IMPORTANT |
| **LLL(MV)** | | | | | | | | | | | | |
| 3 | randomised trials and observational studies | serious^a^ | not serious | not serious | serious^c^ | none | 262 | 469 | - | SMD **0.11 higher** (0.31 lower to 0.53 higher) | ⨁⨁◯◯ Low^a,c^ | IMPORTANT |
| **Restenosis** | | | | | | | | | | | | |
| 4 | randomised trials and observational studies | serious^a^ | not serious | not serious | not serious | none | 6/118 (5.1%) | 33/121 (27.3%) | **OR 0.13** (0.05 to 0.34) | **226 fewer per 1,000** (from 254 fewer to 160 fewer) | ⨁⨁⨁◯ Moderate^a^ | CRITICAL |
| **MI** | | | | | | | | | | | | |
| 8 | randomised trials and observational studies | serious^a^ | not serious | not serious | not serious | none | 29/845 (3.4%) | 42/969 (4.3%) | **OR 0.93** (0.57 to 1.53) | **3 fewer per 1,000** (from 18 fewer to 22 more) | ⨁⨁⨁◯ Moderate^a^ | CRITICAL |
| **TVR** | | | | | | | | | | | | |
| 5 | randomised trials and observational studies | serious^a^ | not serious | not serious | serious^c^ | none | 24/561 (4.3%) | 20/465 (4.3%) | **OR 1.34** (0.72 to 2.48) | **14 more per 1,000** (from 12 fewer to 57 more) | ⨁⨁◯◯ Low^a,c^ | CRITICAL |

**CI:** confidence interval; **OR:** odds ratio; **SMD:** standardised mean difference

#### Explanations

a. Downgraded by one level (-1). The pooled analysis integrated both randomized controlled trials (RCTs) and non-randomized observational studies. The inherent confounding bias from observational designs and the lack of blinding in the device-based RCTs attenuate the overall certainty.

b. Downgraded by two levels (-2) for SB LLL due to extreme and unexplained statistical heterogeneity (I² = 96%). The variation is likely driven by differing DCB brands, lesion preparation techniques, and follow-up durations.

c. Downgraded by one level (-1). The 95% confidence interval is notably wide (or crosses the line of no effect for TVR), and the sample size/number of included studies for this specific angiographic/clinical endpoint is relatively small, reducing the precision of the effect estimate.

Note: This supplementary file includes:

Additional figures and tables

All content supports the findings discussed in the main manuscript.
